# Very preterm, very low birth weight infants not admitted to the neonatal intensive care unit, National Vital Statistics Surveillance Data, United States 2021

**DOI:** 10.1371/journal.pone.0328916

**Published:** 2025-08-12

**Authors:** Madison Levecke, Carla L. DeSisto, Lindsay S. Womack, Ekwutosi M. Okoroh, Shanna Cox, Charlan D. Kroelinger, Wanda D. Barfield

**Affiliations:** 1 Division of Reproductive Health, Centers for Disease Control and Prevention, Atlanta, Georgia, United States of America; 2 Oak Ridge Institute for Science and Education, Oak Ridge, Tennessee, United States of America; 3 United States Public Health Service, Commissioned Corps, Rockville, Maryland, United States of America; Canakkale Onsekiz Mart University School of Medicine, TÜRKIYE

## Abstract

The objective of this analysis is to examine characteristics of very preterm (VPT), very low birth weight (VLBW) infants not admitted to neonatal intensive care units (NICU). In this cross-sectional study assessing VPT (<32 weeks gestation) and VLBW (<1500 grams) infants, we used birth records from the National Vital Statistics System, 2021. Crude and adjusted prevalence ratios (aPR) with modified Poisson regression models were used to calculate prevalence of infants not admitted to the NICU by selected characteristics. Among 38,693 VPT, VLBW infants, 10% were not admitted to the NICU. In the adjusted model, characteristics associated with a higher prevalence of not being admitted to the NICU compared with analytical reference groups included non-Hispanic Native Hawaiian/Other Pacific Islander (aPR = 1.61;95% confidence interval [CI]:1.13–2.29), gestational age 22–24 weeks (aPR = 1.17;CI:1.08–1.26), vaginal delivery (aPR = 1.83;CI:1.73–1.94), and 5-minute Apgar score of 0–3 (aPR = 3.48;CI:3.18–3.82). Exploration of reasons infants were not admitted to the NICU may elucidate strategies to address barriers.

## Introduction

Risk-appropriate care, referred to as perinatal regionalization or perinatal regionalized care, improves health outcomes for infants by providing care in facilities with the personnel and services capable of meeting their health needs, such as provision of care in a neonatal intensive care unit (NICU) [1–3]. NICU care is known to be highly effective in improving outcomes of very preterm newborns [4], yet study results indicate that not all newborns are admitted [5]. Risk-appropriate care of neonates is defined by the American Academy of Pediatrics (AAP) levels of neonatal care policy statements [6,7]. In the most recent AAP levels of neonatal care policy statement, published in 2012 [7] and reaffirmed most recently in 2022 [8], four hospital levels are defined. NICU admission and services are recommended in the AAP policy for Level III hospitals to provide care for infants born <32 weeks gestation or weighing <1500 g and infants with critical illness, and for Level IV hospitals to provide care for the most complex and critically ill newborn infants [6–8].

Evidence has demonstrated improved neonatal and posthospital discharge survival among very preterm (VPT) and very low birth weight (VLBW) infants born in hospitals with Level III+ NICUs (i.e., Level III or IV) [4]. Development of antenatal corticosteroid therapy, early postnatal surfactant therapy, and NICU admission to facilities equipped to provide continuous, comprehensive care for VLBW infants have led to decreases in overall preterm infant mortality rates [9]. AAP recommends that VLBW and/or VPT infants be delivered at Level III+ NICUs, unless this is precluded by the mother’s medical condition or geographic constraints [7].

An analysis of 2006 birth certificate data from 19 states found that overall, only 77.3% of VLBW infants were admitted to the NICU [5]. That study also found NICU admission of VLBW infants varied by state and by race/ethnicity. In multivariable analysis, preterm delivery, multiple gestation, and cesarean delivery were associated with higher prevalence of NICU admission among VLBW infants. A more recent study analyzed trends in NICU admission rates during 2008–2018 and found that NICU admission rates have increased overall and among each racial/ethnic category examined [10]. A recent cross-sectional study published using Vermont Oxford Network (VON) Level III and IV NICU member hospitals concluded that 24.9% of infants born at 22 weeks survived, while a larger proportion of infants survived as gestational age increased with 82.1% born at 25 weeks surviving [11]. A recent case-control study reported the characteristics of pregnant women and infants who did not receive interventions during delivery (i.e., 22–23 weeks gestation) [12]. The authors found that over half of deliveries at 22 weeks and more than 1 in 10 deliveries at 23 weeks gestation received no interventions (e.g., cesarean delivery, maternal transfer, antenatal corticosteroid administration, NICU admission, surfactant administration, antibiotic administration, or assisted ventilation) [12]. In the analysis, sociodemographic characteristics were associated with receiving no neonatal interventions [12].

Additional factors that may impact NICU admission are provider counseling for shared decision making with families not to admit to a NICU [13], as well as the desire to integrate maternal and neonatal care to decrease post-delivery separation [1,14]. Neonatologists providing counseling for parents expecting an extremely preterm (<28 weeks) or extremely LBW (<1,000 grams) infant may include information such as presence of severe congenital anomalies, likelihood of survival, future quality of life, pain management, and parental wishes as considerations that inform decisions on plans of care [13]. Providers can balance clinical issues with parental preference when making decisions about management of extremely preterm infants depending on both maternal and infant interests [15]. Parental participation, such as skin-to-skin holding and integration of families into caregiving to support high-risk infants, is an important part of developmental-based interventions for preterm infants in the NICU [1]. Improving clinical communication and shared decision-making with families may be the most important for improving outcomes and increasing survivability among infants born from gestational ages of 22–25 weeks [15].

In the present study, we examined the characteristics of VPT, VLBW infants not admitted to the NICU in 2021 and clinical interventions by NICU admission status.

## Materials and methods

**Fig 1:**
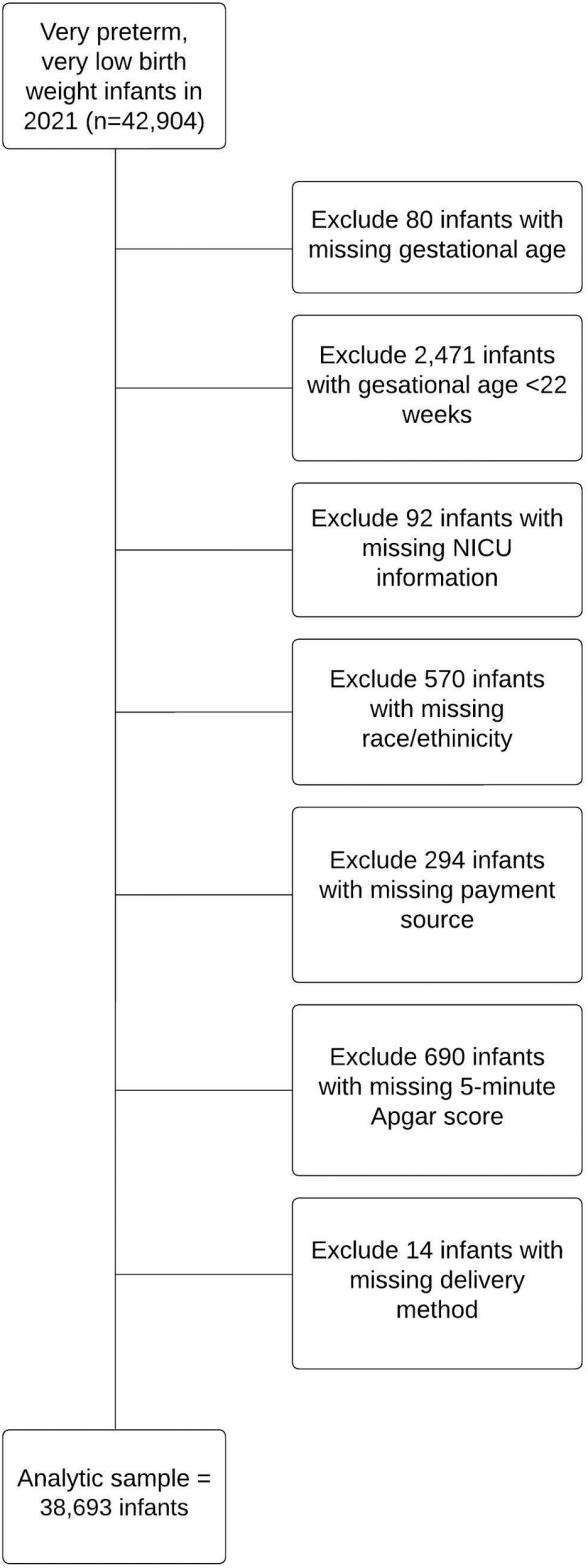
Flow chart demonstrating creation of final analytic data set, National Vital Statistics System, 2021.

This was a retrospective, cross-sectional analysis of one year of birth records from the National Vital Statistics System. Live births from all 50 states in the U.S. and the District of Columbia during January 1 to December 31, 2021, were included. We included infants who were both VPT (gestational age < 32 weeks) and VLBW (<1500 grams). We used the obstetric estimate of gestation at delivery as the gestational age variable [16]. We excluded infants born at <22 weeks because 22 weeks of gestation is clinically accepted as the lower threshold of viability (6%) [17]. We also excluded an additional 4%of infants with missing information on birth weight, gestational age, NICU admission, payment source, maternal race/ethnicity, delivery method, or 5-minute Apgar score (Fig 1).

Our primary outcome of interest was NICU admission, defined as “admission into a facility or unit staffed and equipped to provide continuous mechanical ventilatory support for the newborn” [18]. Birth certificate records do not delineate NICU by level, as the data field is a check box for NICU admission only; therefore, NICU admission status was examined as a binary ‘Yes/No’ variable in our dataset. We examined the distribution of selected characteristics (sex, maternal age, payment source, maternal race/ethnicity, gestational age, plurality, delivery method, and 5-minute Apgar score) by NICU admission status. We calculated chi-square tests to assess differences in distribution of characteristics by NICU admission status.

We calculated the prevalence of NICU admission by each characteristic of interest. We then calculated crude and adjusted prevalence ratios using Poisson regression models with a robust error variance to assess the prevalence of infants not admitted to the NICU by each characteristic [19,20]. Adjusted models included all characteristics of interest.

Finally, we examined clinical interventions by NICU admission status: infant transfer to a different facility within 24 hours, assisted ventilation required immediately after delivery, assisted ventilation required for more than 6 hours, newborn given surfactant replacement therapy, and antenatal corticosteriods for fetal lung maturation received by the mother prior to delivery. We calculated chi-square tests to assess differences in receipt of clinical interventions by NICU admission status.

All analyses were performed in SAS version 9.4 (SAS Institute Inc., Cary, NC). Statistical significance was set at alpha < 0.05. The birth certificate data are available from the National Center for Health Statistics, National Vital Statistics System downloadable at: NVSS - Datasets and Related Documentation for Birth Data. We accessed the data on October 1, 2023, to complete the analysis. This activity was reviewed by the Centers for Disease Control and Prevention (CDC). Because this was a secondary analysis of publicly available, deidentified vital records data this study was not considered human subjects research and did not require approval by the Institutional Review Board at the CDC or participant informed consent.

## Results

Of the 38 693 VPT, VLBW infants born in 2021, 1 in 10 were not admitted to the NICU and 89.9% were admitted to the NICU (Table 1). Demographic information (i.e., infant sex, maternal age, payment source, race and ethnicity) and clinical characteristics (i.e., gestational age, plurality, method of delivery, 5-minute APGAR score) are described.

**Table 1:** Distribution of selected characteristics among very preterm, very low birth weight infants admitted and not admitted to the neonatal intensive care unit, National Vital Statistics System, 2021 (N = 38,693).

	Total N (%)	No NICU Admission N (%)	NICU Admission N (%)	p-value
**Overall**	38,693	3,921 (10.1)	34,772 (89.9)	
**Infant sex**				0.74
Female	19,115 (49.4)	1,947 (49.7)	17,168 (49.4)	
Male	19,578 (50.6)	1,974 (50.3)	17,604 (50.6)	
**Maternal age, years**				0.23
< 20	1,872 (4.8)	209 (5.3)	1,663 (4.8)	
20–29	17,120 (44.2)	1,750 (44.6)	15,370 (44.2)	
30–39	17,643 (45.6)	1,772 (45.2)	15,871 (45.6)	
40+	2,058 (5.3)	190 (4.8)	1,868 (5.4)	
**Payment source**				0.001
Medicaid	19,169 (49.5)	1,984 (50.6)	17,185 (49.4)	
Private	16,901 (43.7)	1,624 (41.4)	15,277 (43.9)	
Self-pay	1,273 (3.3)	170 (4.3)	1,103 (3.2)	
Other	1,350 (3.5)	143 (3.6)	1,207 (3.5)	
**Maternal race and Hispanic origin**				< 0.0001
Non-Hispanic White	14,734 (38.1)	1,393 (35.5)	13,341 (38.4)	
Non-Hispanic Black	11,846 (30.6)	1,201 (30.6)	10,645 (30.6)	
Hispanic	8,857 (22.9)	936 (23.9)	7,921 (22.8)	
Non-Hispanic Asian	1,890 (4.9)	220 (5.6)	1,670 (4.8)	
Non-Hispanic American Indian/Alaska Native	261 (0.7)	35 (0.9)	226 (0.6)	
Non-Hispanic Native Hawaiian or Other Pacific Islander	125 (0.3)	23 (0.6)	102 (0.3)	
Non-Hispanic, more than one race	980 (2.5)	113 (2.9)	867 (2.5)	
**Gestational age, weeks**				< 0.0001
22–24	7,038 (18.2)	1,412 (36.0)	5,626 (16.2)	
25–27	11,844 (30.6)	932 (23.8)	10,912 (31.4)	
28–31	19,811 (51.2)	1,577 (40.2)	18,234 (52.4)	
**Plurality**				< 0.0001
Singleton	29,754 (76.9)	3,276 (83.6)	26,478 (76.1)	
Twin	8,132 (21.0)	614 (15.7)	7,518 (21.6)	
Triplet or more	807 (2.1)	31 (0.8)	776 (2.2)	
**Delivery method**				< 0.0001
Vaginal	10,341 (26.7)	1,847 (47.1)	8,494 (24.4)	
Cesarean	28,352 (73.3)	2,074 (52.9)	26,278 (75.6)	
**5-minute APGAR score**				< 0.0001
0–3	4,758 (12.3)	1,563 (39.9)	3,195 (9.2)	
4–6	7,905 (20.4)	570 (14.5)	7,335 (21.1)	
7–8	17,283 (44.7)	1,112 (28.4)	16,171 (46.5)	
9–10	8,747 (22.6)	676 (17.2)	8,071 (23.2)	

NICU, neonatal intensive care unit.

In the adjusted model, factors associated with a higher prevalence of not being admitted to the NICU included non-Hispanic NHOPI (adjusted prevalence ratio [aPR]= 1.61, 95% confidence interval [CI]: 1.13–2.29), as compared to non-Hispanic White maternal race; gestational age 22–24 weeks (aPR = 1.17, 95% CI: 1.08–1.26), as compared to 28–31 weeks; vaginal delivery (aPR = 1.83, 95% CI: 1.73–1.94), as compared to Cesarean delivery; and 5-minute Apgar score of 0–3 (aPR = 3.48, 95% CI: 3.18–3.82), as compared to 9–10 (Table 2). Factors associated with a lower prevalence of not being admitted to the NICU included gestational age of 25–27 weeks (aPR = 0.84, 95% CI: 0.77–0.90), as compared to 28–31 weeks; twin birth or more (aPR = 0.76, 95% CI: 0.70–0.82), as compared to singleton birth; and 5-minute Apgar score of 7–8 (aPR = 0.85, 95% CI: 0.78–0.94), as compared to a score of 9–10.

**Table 2:** Crude and adjusted prevalence ratios for very preterm, very low birth weight infants not admitted to neonatal intensive care units, by selected characteristics, National Vital Statistics System, 2021 (N = 38,693).

	Total not admitted to NICU N (row %)	Total admitted to NICU N (row %)	Crude prevalence ratio (95% CI)	Adjusted^1^ prevalence ratio (95% CI)
**Infant sex**				
Female	1,947 (10.2)	17,168 (89.8)	Ref	Ref
Male	1,974 (10.1)	17,604 (89.9)	0.99 (0.93, 1.05)	0.95 (0.90, 1.00)
**Maternal age, years**				
< 20	209 (11.2)	1,663 (88.8)	1.11 (0.97, 1.27)	0.94 (0.82, 1.06)
20–29	1,750 (10.2)	15,370 (89.8)	1.02 (0.96, 1.08)	0.98 (0.92, 1.04)
30–39	1,772 (10.0)	15,871 (90.0)	Ref	Ref
40+	190 (9.2)	1,868 (90.8)	0.92 (0.80, 1.06)	0.97 (0.85, 1.12)
**Payment source**				
Medicaid	1,984 (10.4)	17,185 (89.6)	1.08 (1.01, 1.15)	1.01 (0.95, 1.07)
Private	1,624 (9.6)	15,277 (90.4)	Ref	Ref
Self-pay/Other	313 (11.9)	2,310 (88.1)	1.24 (1.11, 1.39)	1.06 (0.95, 1.18)
**Maternal race and Hispanic origin**				
Non-Hispanic White	1,393 (9.5)	13,341 (90.5)	Ref	Ref
Non-Hispanic Black	1,201 (10.1)	10,645 (89.9)	1.07 (1.00, 1.15)	0.99 (0.93, 1.07)
Hispanic	936 (10.6)	7,921 (89.4)	1.12 (1.03, 1.21)	1.03 (0.96, 1.12)
Non-Hispanic American Indian/Alaska Native	35 (13.4)	226 (86.6)	1.42 (1.04, 1.94)	1.24 (0.93, 1.66)
Non-Hispanic Asian	220 (11.6)	1,670 (88.4)	1.23 (1.08, 1.41)	1.12 (0.99, 1.27)
Non-Hispanic Native Hawaiian or Other Pacific Islander	23 (18.4)	102 (81.6)	1.95 (1.34, 2.82)	**1.61 (1.13, 2.29)**
More than one race	113 (11.5)	867 (88.5)	1.22 (1.02, 1.46)	1.15 (0.97, 1.36)
**Gestational age, weeks**				
22–24	1,412 (20.1)	5,626 (79.9)	2.52 (2.36, 2.69)	**1.17 (1.08, 1.26)**
25–27	932 (7.9)	10,912 (92.1)	0.99 (0.92, 1.07)	**0.84 (0.77, 0.90)**
28–31	1,577 (8.0)	18,234 (92.0)	Ref	Ref
**Plurality**				
Singleton	3,276 (11.0)	26,478 (89.0)	Ref	Ref
Twin or more	645 (7.2)	8,294 (92.8)	0.66 (0.60, 0.71)	**0.76 (0.70, 0.82)**
**Delivery method**				
Vaginal	1,847 (17.9)	8,494 (82.1)	2.44 (2.30, 2.59)	**1.83 (1.73, 1.94)**
Cesarean	2,074 (7.3)	26,278 (92.7)	Ref	Ref
**5 minute APGAR score**				
0–3	1,563 (32.8)	3195 (67.2)	4.25 (3.91, 4.62)	**3.48 (3.18, 3.82)**
4–6	570 (7.2)	7,335 (92.8)	0.93 (0.84, 1.04)	0.92 (0.82, 1.03)
7–8	1,112 (6.4)	16,171 (93.6)	0.83 (0.76, 0.91)	**0.85 (0.78, 0.94)**
9–10	676 (7.7)	8,071 (92.3)	Ref	Ref

Abbreviations: NICU, neonatal intensive care unit.

^1^Adjusted for all characteristics of interest listed in this table.

Infants not admitted to the NICU during their birth hospitalization were more likely to be transferred than infants admitted to the NICU (13.3% vs. 10.7%, p < 0.0001, Table 3). Infants not admitted to the NICU during their birth hospitalization were less likely to receive any assisted ventilation (18.8% vs. 62.6%), receive assisted ventilation for > 6 hours (4.4% vs. 41.2%), receive surfactant (4.0% vs. 23.0%), or have their mother receive antenatal steroids (26.5% vs. 53.0%), compared with infants admitted to the NICU (p < 0.0001 for all).

**Table 3:** Clinical interventions among very preterm, very low birth weight infants admitted and not admitted to the Neonatal Intensive Care Unit, National Vital Statistics System, 2021 (N = 38,693).

	No NICU Admission	NICU Admission	p-value
**Infant transferred**			< 0.0001
No	3,397 (86.6)	31,032 (89.2)	
Yes	522 (13.3)	3,719 (10.7)	
Unknown	2 (0.1)	21 (0.1)	
**Assisted ventilation**			< 0.0001
No	3,185 (81.2)	13,014 (37.4)	
Yes	736 (18.8)	21,758 (62.6)	
**Assisted ventilation > 6 hrs**			< 0.0001
No	3,749 (95.6)	20,459 (58.8)	
Yes	172 (4.4)	14,313 (41.2)	
**Infant received surfactant**			< 0.0001
No	3,765 (96.0)	26,762 (77.0)	
Yes	156 (4.0)	8,010 (23.0)	
**Maternal receipt of antenatal steroids**			< 0.0001
No	2,877 (73.4)	16,337 (47.0)	
Yes	1,040 (26.5)	18,424 (53.0)	
Unknown	4 (0.1)	11 (0.0)	

NICU, neonatal intensive care unit.

## Discussion

In 2021, NICU admission for VPT, VLBW infants varied by maternal race/ethnicity, gestational age, plurality, delivery method, and 5-minute Apgar score. Additionally, we found that 90% of VPT, VLBW infants were admitted to the NICU. This finding exceeded the previous national benchmark measure listed in Healthy People 2020, which focused on more specialized facilities likely to have a NICU. Healthy People Maternal, Infant, and Child Health objective 33 was to increase the proportion of VLBW infants born at Level III hospitals or subspecialty perinatal centers from 75.0% in 2003–2006 to 83.7% by 2020 [21]. Continued surveillance of NICU admission status offers opportunity to monitor whether the prevalence of risk-appropriate care practices is improving, especially among VLBW and/or VPT infants who experience disproportionate morbidity and mortality.

We found that infants born at 22–24 weeks had about 1.2 times the prevalence of not being admitted to the NICU compared with infants born at 28–31 weeks. Similarly, we found that infants with a 5-minute Apgar score of 0–3 had more than three times the prevalence of not being admitted to the NICU compared with infants with a 5-minute Apgar score of 9–10. An AAP clinical report for infants under 25 weeks states, “…decision-making regarding the delivery room management can be individualized and family centered, taking into account known fetal and maternal conditions and risk factors as well as parental beliefs regarding the best interest of the child…When a decision is made not to resuscitate a newborn infant, comfort care is appropriate, as is encouraging the family to spend time with the dying/deceased newborn infant. Providing religious, psychosocial, and/or palliative care support may assist families at this difficult time” [17].

We found that compared with infants with non-Hispanic White mothers, infants with non-Hispanic NHOPI had a higher prevalence of not being admitted to the NICU in the adjusted model. Previous studies that have examined NICU admission among VLBW infants have only reported on non-Hispanic White, non-Hispanic Black, and Hispanic groups [5,10]. A National Vital Statistics Report found that NHOPI infants had almost twice the infant mortality rate and more than three times the post-neonatal mortality rate compared with non-Hispanic White infants in 2018 [22]. Additionally, a recent Kaiser Family Foundation (KFF) report documented that NHOPI women compared with White women have higher rates of both preterm and low birthweight births, and received delayed or no prenatal care [23]. These differences may be indicators of health coverage and access to and use of care issues for this population, though NHOPI infant outcomes are historically under-researched and underreported [24,25]. In our analysis, there was no statistical difference between infants of non-Hispanic White mothers and infants of non-Hispanic Black mothers for NICU admission in the adjusted model. This is consistent with an analysis of temporal trends of NICU admission among VLBW infants during 2008–2018, which found a very small difference (although statistically significant) in overall risk-adjusted NICU admission when comparing infants with non-Hispanic Black mothers to infants with non-Hispanic White mothers [10]. Similarly, an analysis of VLBW infants in 2006 found no difference in the adjusted prevalence of NICU admission comparing infants with non-Hispanic Black mothers to infants with non-Hispanic White mothers [5]. More research is needed to better understand patterns of NICU admission that vary by race/ethnicity to support delivery of care for all mothers and infants.

In this analysis, infants born vaginally had increased prevalence of not being admitted to the NICU, relative to infants born via cesarean delivery. Infants delivered by cesarean might have medical indications for this delivery method (e.g., complex congenital anomalies, fetal distress), and those indications can increase the likelihood of NICU admission [5]. Further, we found that multiples had decreased prevalence of not being admitted to the NICU relative to singleton infants. Multiple gestation infants may need more specialized care [5]. A previous study of VLBW infants using 2006 birth certificate data also found that multiples and infants born via cesarean delivery were positively associated with NICU admission [5].

In our study, mothers of infants not admitted to the NICU were less likely to have received antenatal steroids compared with mothers of infants admitted to the NICU. Antenatal steroids, given primarily to induce pulmonary maturity and surfactant release and decrease respiratory distress syndrome in the infant [26], is associated with improved outcomes, especially in infants born at younger gestational ages [26–29]. A complete course of antenatal steroids is defined as two intramuscular doses of betamethasone given 12–24 hours apart or four intramuscular doses of dexamethasone given 12 hours apart [30,31]. However, preterm infants may be born prior to the completion of the course of maternal antenatal steroids [32]. Our data set does not contain information about whether mothers received the full course of antenatal steroids prior to delivery [18].

We found that infants not admitted to the NICU were more likely to be transferred compared with infants admitted to the NICU. Our data set does not contain information on the clinical interventions received after hospital transfer, including NICU admission at the receiving hospital. Although maternal transport to an appropriate level facility during the antenatal period is considered ideal so that VPT or VLBW infants are born in facilities with Level III+ NICUs [33], neonatal and obstetrical complications can be unpredictable, and the opportunity to transport the neonate after delivery is critical to providing the infant with the most appropriate care [34,35].

Persistent barriers to admission of VPT, VLBW infants to Level III+ NICUs include underuse of NICU care for VLBW infants [36], distances between patients and the nearest specialized medical center [37], de-regionalization and deregulation of risk-appropriate care [35,38–41], and gaps in state policies outlining the capabilities and specialty staffing of NICUs [42]. However, several state and national efforts continue to improve risk-appropriate NICU admissions. For example, to disseminate evidence-based practices for risk-appropriate neonatal care, many states and facilities are participating in continuous quality improvement initiatives through Perinatal Quality Collaboratives, or networks of providers in facilities testing and implementing best clinical practices [43,44]. Additionally, AAP conducts a NICU verification program; this program is designed to review NICUs to verify whether they meet the standards for a specific level of neonatal care [45]. This program aims to improve outcomes for high-risk infants by ensuring every high-risk newborn receives care in a facility with the personnel and resources appropriate for the infant’s needs [45], and provided evidence to establish risk-appropriate neonatal care standards for Level II, III, and IV facilities to improve the quality and consistency of care aligned with neonatal levels of care guidelines [14]. The CDC developed the Levels of Care Assessment Tool (CDC LOCATe^TM^) [46], which provides standardized assessments of neonatal and maternal levels of care in alignment with the guidance from AAP and the American College of Obstetricians and Gynecologists/Society for Maternal Fetal Medicine [7,47]. These standardized LOCATe^TM^ assessments can help organizations, such as state health departments and Perinatal Quality Collaboratives, identify opportunities to improve risk-appropriate care, including the care provided to VPT, VLBW infants.

This study is subject to several limitations. First, our data set does not include the neonatal level of care of individual delivery hospitals, as the data field is a check box for NICU admission only. Related to this, NICU admission, our primary outcome of interest, could have been incorrectly reported on the infant birth certificate. Although the birth certificate form specifies the definition of NICU admission [18], defined as, ‘admission into a facility or unit staffed and equipped to provide continuous mechanical ventilatory support for a newborn’ [48], and AAP defines a NICU as being in a Level III+ facility, states may vary in their definitions, criteria, and regulatory status [42,49,50]. Specifically, the number of midlevel NICUs or special care nurseries and neonatologists practicing outside of Level III+ facilities has increased [38,39,41]. Therefore, this analysis does not specifically represent Level III+ NICUs; rather, it only represents NICUs more generally. At the same time, there are known inaccuracies in the documentation of NICU admissions on the birth certificates with variation by state [51–53]. A study comparing birth certificate data with information abstracted from hospital medical records in two states found the sensitivity of the NICU field on the birth certificate was 45.1% in one state and 95.1% in the other state [51], and a follow-up study reported that the sensitivity was 49.3% in New York City [52]. A study from California also found birth certificate data under-reported NICU admissions [53]. NICU admissions may also be under-ascertained in this study if infants were admitted to a NICU after transfer to another hospital; however, a recent study using birth certificate data conducted a sensitivity analysis assuming transferred infants were all admitted to the NICU and found little change in the prevalence of NICU admission [10]. Further, our data, including maternal and infant characteristics and clinical interventions, are subject to misclassification or reporting bias if information was not correctly added to the infant birth certificate or misinterpreted by a third-party during data entry. For example, the variable for antenatal corticosteroids may have poor sensitivity or may be incomplete [52,54], as timing of steroid administration is not captured on the birth certificate [54]; one study concluded most women gave birth more than 14 days after administration [55]. Additionally, 10% of our sample was excluded due to missing data, which may limit the generalizability of these findings to all VPT, VLBW infants. Finally, we do not have information about what antenatal counseling decisions were made, nor whether resuscitation or comfort care were initiated which could account for joint decision-making between parents and providers on subsequent NICU admission. Data on subsequent infant outcomes, such as mortality, were not included. Data on infant mortality are found on death certificates, separate from infant live birth certificates. To study infant outcomes, births that occur in 2021 are linked with death records up to one year following live birth, and the final, linked birth and infant death file for all deaths occurring in 2022 was unavailable at the time of this analysis [56]. It may be that information included on infant deaths can inform interpretation of the variation in non-NICU admission. Related, we were unable to disaggregate data by week of gestational age due to lack of statistical power in our modeling, which could impact results. Future research could aggregate data over multiple years to examine results by each week of gestational age.

Given our study cannot infer causality, future research could also examine reasons for NICU admission variability. Additional factors not assessed in this study, such as NICU availability, are not available on the birth certificate. While this analysis aimed to provide a national snapshot based on individual characteristics, birth certificate data could be used to access patterns by rurality, region, and state specific analyses to highlight geographic patterns in NICU admission. As neonatal care policies vary by state [2], conducting individual state analyses may provide additional reasons for non-NICU admission [17]. Further, it is known that birth certificate data may not accurately report NICU admission [51–53]; therefore, assessment of reasons for non-NICU admissions, such as availability of NICU at facility of delivery, transfers to higher level of care, or verification of NICU admission, can be informed by use of hospital medical records. Medical record data are considered the gold standard for retrospective hospital-based studies and for assessing quality of patient care and specific clinical outcomes [57].

## Conclusion

Overall, NICU admission was high among VPT, VLBW infants; however, it varied by maternal race/ethnicity, gestational age, plurality, delivery method, and 5-minute Apgar score. As noted, many limitations inherent to the dataset used and misclassification of NICU admission may have impacted study results. The goal of regionalized perinatal care is to ensure that mothers with high-risk pregnancies—such as mothers delivering VPT, VLBW infants—give birth in Level III+ facilities with access to NICU services immediately following delivery. Risk-appropriate care in NICUs for VPT, VLBW infants is critical for improving neonatal and posthospital discharge survival. Continued surveillance, addressing barriers, and supporting facilitators to NICU admission for VPT, VLBW infants could improve perinatal care infrastructure and practice in the United States.
